# Regulation of sonic hedgehog-*GLI1 *downstream target genes *PTCH1, Cyclin D2, Plakoglobin, PAX6 and NKX2.2 *and their epigenetic status in medulloblastoma and astrocytoma

**DOI:** 10.1186/1471-2407-10-614

**Published:** 2010-11-08

**Authors:** Mehdi H Shahi, Mohammad Afzal, Subrata Sinha, Charles G Eberhart, Juan A Rey, Xing Fan, Javier S Castresana

**Affiliations:** 1 Brain Tumor Biology Unit-CIFA, University of Navarra School of Sciences, Pamplona, Spain; 2 Department of Zoology, Aligarh Muslim University, Aligarh, India; 3 Department of Biochemistry, All India Institute of Medical Sciences, New Delhi, India; 4 Department of Pathology, Johns Hopkins University School of Medicine, Baltimore, MD, USA; 5 Research Unit, La Paz University Hospital, Madrid, Spain; 6 Department of Neurosurgery, University of Michigan Medical School, Ann Arbor, MI, USA

## Abstract

**Background:**

The Sonic hedgehog (Shh) signaling pathway is critical for cell growth and differentiation. Impairment of this pathway can result in both birth defects and cancer. Despite its importance in cancer development, the Shh pathway has not been thoroughly investigated in tumorigenesis of brain tumors. In this study, we sought to understand the regulatory roles of *GLI1*, the immediate downstream activator of the Shh signaling pathway on its downstream target genes *PTCH1*, *Cyclin D2*, *Plakoglobin*, *NKX2.2 *and *PAX6 *in medulloblastoma and astrocytic tumors.

**Methods:**

We silenced *GLI1 *expression in medulloblastoma and astrocytic cell lines by transfection of siRNA against *GLI1*. Subsequently, we performed RT-PCR and quantitative real time RT-PCR (qRT-PCR) to assay the expression of downstream target genes *PTCH1, Cyclin D2, Plakoglobin, NKX2.2 *and *PAX6*. We also attempted to correlate the pattern of expression of *GLI1 *and its regulated genes in 14 cell lines and 41 primary medulloblastoma and astrocytoma tumor samples. We also assessed the methylation status of the *Cyclin D2 *and *PTCH1 *promoters in these 14 cell lines and 58 primary tumor samples.

**Results:**

Silencing expression of *GLI1 *resulted up-regulation of all target genes in the medulloblastoma cell line, while only *PTCH1 *was up-regulated in astrocytoma. We also observed methylation of the *cyclin D2 *promoter in a significant number of astrocytoma cell lines (63%) and primary astrocytoma tumor samples (32%), but not at all in any medulloblastoma samples. *PTCH1 *promoter methylation was less frequently observed than *Cyclin D2 *promoter methylation in astrocytomas, and not at all in medulloblastomas.

**Conclusions:**

Our results demonstrate different regulatory mechanisms of Shh-*GLI1 *signaling. These differences vary according to the downstream target gene affected, the origin of the tissue, as well as epigenetic regulation of some of these genes.

## Background

The Sonic hedgehog (Shh) signaling pathway is crucial for embryonic development and is involved in the fate of many tissues during organogenesis, including the central nervous system [1-4]. Additionally, the Shh signaling pathway has been implicated in stem cell renewal [5] as well as in the development of tumors such as medulloblastoma [1,6], prostate cancer [2,7,8] colorectal carcinoma [9], and glioma [10].

This pathway is initiated by ligation of the Shh protein with its receptor *PTCH1 *on a target cell. Its binding relieves the inhibition of *Smoothened *(*SMO*) by *PTCH1*. The active *SMO *enters the cytoplasm and activates *GLI1. GLI1 *is then phosphorylated by the fused serine/threonine kinase [11] and Costal-2, a kinase-like cytoplasmic protein (Cos2) [12], and finally enters the nucleus where it acts as a transcriptional regulator on the promoter regions of different target genes. Downstream target genes of *GLI1 *are *PTCH1*, *Wnt*, as well as other genes such as *Cyclin D2 *and *Plakoglobin*. These genes have consensus sequences for GLI1 binding in their 5'upstream promoter region. Reports have shown that *Cyclin D2 *is up-regulated while *Plakoglobin *is down-regulated by *GLI1 *in epithelial transformed cell lines [13].

Direct regulation of *PAX6 *and *NKX2.2 *by *GLI1 *has not yet been reported. However, Shh signaling regulates expression of these genes during the early embryonic stage of neuronal development. *PAX6 *is down-regulated and *NKX2.2 *is up-regulated by Shh in a dose dependent manner [14]. *PAX6 *also inhibits glioma invasion and acts as a tumor suppressor gene [15,16]. *NKX2.2 *is expressed in low-grade gliomas but not in high-grade gliomas [17]. However, direct regulation of these genes by *GLI1 *has not been studied in major brain tumors such as medulloblastomas and astrocytomas.

Reports have shown that medulloblastomas arise from granule cell progenitors (GCP) \ [18,19] by two postulated mechanisms: either excessive signaling stimulating GCP proliferation, or absence of appropriate signals for GCPs to stop dividing [20]. The role of Shh signaling pathway in medulloblastoma tumor development had its origins in the Gorlin syndrome, also known as the basal cell carcinoma syndrome, an autosomal dominant disease with an incidence of about 1 in 50,000 live births. Gorlin syndrome is caused by *PTCH1 *mutations in about 85% of cases. At least 25% of medulloblastoma sporadic tumors show *PTCH1 *mutations [21]. The major focus of medulloblastoma tumor research is now based upon GCPs as a source of medulloblastoma, although certain populations of neural stem cells (NSCs) are gaining importance [22,23]. The role of Shh signaling is less well studied in astrocytomas. Additionally, the involvement of *GLI1 *downstream target genes such as *PTCH1, Cyclin D2, Plakoglobin, NKX2.2 *and *PAX6 *have not been studied in either tumor.

In our study, we have silenced expression of *GLI1 *using a small interfering RNA (siRNA), followed by the determination of gene expression patterns of *PTCH1, Cyclin D2, Plakoglobin, NKX2.2 *and *PAX6 *in 14 cell lines and 41 primary medulloblastoma and astrocytoma tumor samples.

Our observations on the expression of *GLI1, Cyclin D2, and PTCH1 *in astrocytoma cell lines as well as tumor samples revealed a significant proportion of samples showing low or null levels of *Cyclin D2 *and *PTCH1*, even in the presence of high *GLI1 *expression. However, high levels of *GLI1 *correlated with high levels of *Cyclin D2 *and *PTCH1 *in medulloblastoma cell lines and tumor samples.

We also explored the possibility of epigenetic regulation of *Cyclin D2 *and *PTCH1 *in astrocytic cell lines and samples. We used demethylating agents including 5-Aza-2'-deoxycytidine (5-Aza-2'-dC) and the histone deacetylase (HDAC) inhibitor Trichostatin A, to assess reversal of expression of *Cyclin D2 *in astrocytic cell lines wherein the gene is not naturally expressed. To further our understanding of epigenetic regulation, we also monitored the methylation status of the *Cyclin D2 *and *PTCH1 *promoters in 14 cell lines and 58 tumor samples derived from medulloblastomas and astrocytomas. We performed melting curve analysis-methylation assays (MCA-Meth) and melting curve analysis-methylated specific PCR (MCA-MSP) to assess methylation at these genetic loci [24].

Thus, our objectives were two-fold: first, to determine whether *GLI1 *up-regulates any of its downstream effectors; and second, to study potential epigenetic regulation of *PTCH1 *and *Cyclin D2.*

## Methods

### Cell lines

For this study, we chose 6 medulloblastoma cell lines (TE671, PFSK-1, Daoy, TE671c2, D283Med and SK-PN-DW) and 8 high-grade astrocytoma cell lines (U87MG, A172, LN405, SW1783, T98G, SW1088, CCF-STTG1, and GOS-3). The cell lines PFSK-1, Daoy, D238, SK-PN-DW, CCF-STTG1, SW1088 and SW1783 were purchased from the American Type Culture Collection (Manassas, VA, USA). A172, T98G and U87MG were purchased from the European Collection of Cell Culture (Salisbury, Wiltshire, UK). TE671, TE671c2, LN405 and GOS-3 were obtained from the Deutsche Sammlung von Mikroorganismen und Zellkulturen (Braunschweig, Germany). The cell lines were cultured in RPMI L-Glutamax medium (GIBCO-BRL, Gaithersburg, MD, USA), supplemented with 10% fetal bovine serum (FBS), 1% penicillin/streptomycin, 0.1% amphotericin B, and for medulloblastoma cell lines, 10% non-essential amino acids (NEAs). Cells lines were maintained at 37°C in the presence of 5% CO_2_. On attaining 80% confluence, the cells were split using trypsin/EDTA (1×) and plated in new sterile flasks.

### Primary tumor samples

We used 14 primary medulloblastomas (Tables 1 and 2) and 44 primary astrocytomas (Tables 3 and 4). The use of medulloblastoma samples for research purposes was approved by Johns Hopkins Medical Institute (JHMI), USA Institutional Review Board under protocol #99-12-29-05. Similarly, the use of astrocytoma samples was approved by the Ethical Committee of the University of Navarra Medical School, Pamplona, Spain under the protocol #38/2002, February and 04/2/2008. The astrocytomas were of WHO grades I to IV. All samples were snap frozen immediately on resection and stored at -80°C. Genomic DNA, RNA and proteins were extracted from the frozen tissues.

**Table 1:** Promoter methylation and expression correlation of *Cyclin D2 *and *PTCH1 *gene in medulloblastoma cell lines

Cell lines	Cyclin D2 expression	Cyclin D2 promoter hypermethylation by MCA-Meth	Cyclin D2 Hypermethylation by MSP	Cyclin D2 Hypermethylation by both methods	No Hyper Methylation in Cyclin D2 Promoter	PTCH1 Expression in medulloblastoma cell lines	PTCH1 promoter Hypermethylation by MCA-MSP method
**TE671**	++	U	M			++	U

**PFSK-1**	++	M	M	√		++	U

**Daoy**	++	U	U+M			++	U

**TE671c2**	++	U	U+M			+	U

**D283**	++	U	U + M			+	U+M

**SK-PN-DW**	+	U+M	M	√		+	U

-: no expression; +: expression; ++: high expression; √: yes

**Table 2:** Promoter methylation and expression correlation of *Cyclin D2 *and *PTCH1 *gene in medulloblastoma samples

MB samples ID	Patients' Date of Birth (D/M/Y)	Sex	Expression of Cyclin D2 in primary tumor samples	Promoter methylation by MCA-Meth in Cyclin D2	Promoter methylation by MSP in Cyclin D2	No Promoter hypermethylation in Cyclin D2	Expression of PTCH1 in primary tumor samples	Promoter hypermethylation in PTCH1 by MCA-Meth of PTCH1
p3	5/18/1969	M	++	U	U	√	-	U + M

p4	3/9/1987	M	-	U	U + M		+	U

p7	11/27/1987	M	++	U	U	√	+	U

p10	4/15/1971	M	++	U	U	√	nd	U

p11	4/30/1997	F	+	U	U + M		-	U + M

p13	8/21/1995	F	+	U	U + M		nd	U

p14	9/9/1991	F	++	U	U	√	+	U

p17	11/30/1988	M	++	U	U	√	+	U

p20	11/22/1978	M	++	U	U	√	+	U

p21	11/15/1987	M	++	U	U	√	+	U

p29	3/9/1974	M	++	U	U	√	nd	U

p30	6/24/1991	M	++	U	U	√	nd	nd

p23	8/15/1984	F	nd	nd	nd	nd	nd	nd

p52	10/5/1991	M	nd	nd	nd	nd	nd	nd

MB: medulloblastoma; -: no expression; +: expression; ++: high expression; √: yes

**Table 3:** *Cyclin D2 *and *PTCH1 *expression and promoter hypermethylation analysis in astrocytic cell lines

Astrocytic Cell lines	Cyclin D2 expression	Cyclin D2 Expression after 5'aza+TSA	Cyclin D2 promoter hypermethylation analysis by MCA-MSP	Cyclin D2 promoter hypermethylation Analysis by MCA-Meth	Cyclin D2 Hypermethylation by both techniques	No Hypermethylation in Cyclin D2 promoter	PTCH1 expression in glioma cell lines	PTCH1 promoter hypermethylation by MCA-MSP
U87MG	+	+	√	√	√		+/-	

A172	-	+				√	-	

LN405	+	+				√	+	

SW1783	-	+	√				-	

T98G	-	++	√	√	√		++	

SW1088	+	+				√	++	

CCF-STTG-1	-	+	√				++	√

GOS-3	-	++	√	√	√		++	

-: no expression; +: expression; ++: high expression; √: yes

**Table 4:** Expression and promoter hypermethylation analysis of *Cyclin D2 *and *PTCH1 *gene in astrocytic tumor samples

Astrocytic tumor samples Grade and Sample ID	Expression of Cyclin D2 in tumor samples	Methylation by MSP in Cyclin D2 promoter	Methylation by MCA-Meth in Cyclin D2 promoter	Methylation by both techniques in Cyclin D2 promoter	No hypermethylation in Cyclin D2 promoter	Expression of PTCH1 in tumor samples	Methylation of PTCH1 promoter by MCA-Meth
GBM(3)	-	√	√	√		-	√

AIII(4)	++				√	+	

GBM(5)	-				√	+/-	

GBM(6)	-	√				+/-	

AIII(7)	+				√	+/-	

GBM(8)	-	√				++	

GBM(9)	+				√	-	

AIII(10)	-	√				+/-	

GBM(11)	++				√	+/-	

GBM(12)	+				√	+	

GBM(13)	-	√	√	√		+	√

AIII(14)	++		√			-	

GBM(15)	-	√				+/-	

AIII(16)	++	√				+/-	

GBM(17)	-		√			++	

GBM(18)	-	√	√	√		+	

AIII(19)	+		√			+	

AII(20)	+		√			+	

AIII(21)	-	√				-	√

GBM(22)	-		√			-	

GBM(23)	-		√			+	

GBM(24)	-	√	√	√		+	

AI(25)	ND				√	ND	

GBM(26)	ND				√	ND	

GBM(27)	ND				√	ND	

GBM(28)	ND				√	ND	

GBM(29)	ND				√	ND	

GBM(30)	+	√				+	

GBM(31)	ND				√	ND	

GBM(32)	ND				√	ND	

AI(34)	ND				√	ND	

AIII(35)	ND				√	ND	

GBM(36)	ND				√	ND	

GBM(41)	ND				√	ND	

GBM(43)	+	√				-	

GBM(44)	ND				√	ND	

GBM(45)	ND				√	ND	

GBM(46)	ND				√	ND	

GBM(47)	ND				√	ND	

GBM(48)	ND	√				ND	

GBM(49)	ND	√				ND	

AII(50)	++				√	-	

GBM(51)	-	√				-	

GBM(52)	++				√	+	

-:no expression; +/-: low expression; +: expression; ++: high expression; √: yes; nd: not determined

### *GLI1 *siRNA transfection and knock-down

For these experiments, we selected the medulloblastoma cell line Daoy, and the astrocytic cell line U87MG, which were grown in RPMI L-Glutamax medium (GIBCO-BRL, Gaithersburg, MD, USA), supplemented with 2% fetal bovine serum (FBS) at 37°C in the presence of 5% CO_2_. We procured siRNA against the *GLI1 *gene from Stealth™ (Invitrogen, USA) *GLI1 *RNAi: GCACAUACCUGCUUCGGGCAAGAUAU (GLI-HSS104170) and AUAUCUUGCCCGAAGCAGGUAGUGC (GLI-HSS104170), along with a universal negative siRNA to control for non-specific interference by the siRNA. The universal negative siRNA used was a scrambled sequence with medium GC content and does not influence expression of the target gene. The *GLI1 *siRNA and the scrambled siRNA were delivered to the Daoy and U87MG cell lines (100 nM/well), using Lipofectamine™ 2000 as the transfection reagent and Opti-MEM^® ^I reduced serum media as a mixing reagent solution for the siRNA and LipofectamineTM 2000. We used BLOCK-iT™ Fluorescent Oligo tagged with fluorescein to assess and optimize cationic lipid-mediated delivery of siRNA into the Daoy and U87MG cell lines. Following efficient transfection, we extracted RNA from cells transfected with *GLI1 *siRNA, scrambled siRNA, as well as untransfected cells after 72 h. Finally, we assessed efficiency of *GLI1 *silencing in the two cell lines. The extracted RNA was used to assess expression levels of downstream target genes of the Shh pathway such as *PTCH1, Cyclin D2, Plakoglobin, PAX6 *and *NKX2.2.*

### Standard RT-PCR and quantitative real time RT-PCR (qRT-PCR)

We used the QuickPrep Total RNA extraction Kit (Amersham Biosciences, UK) to extract RNA from the two transfected cell lines, Daoy and U87MG. Normal adult brain RNA (Stratagene, Cedar Creek, TX) was used as a control for mRNA expression. A total of 1 μg of RNA was converted to cDNA by the Superscript II RNase H Reverse Transcriptase kit (Invitrogen, Life Technologies, Carlsbad, CA). The cDNA was amplified by standard RT-PCR using oligonucleotides published elsewhere [9]. We compared mRNA expression in *GLI1 *silenced cell lines with cells transfected with scrambled siRNA as well as untransfected cell lines. We confirmed *GLI1 *silencing by quantifying *GLI1 *transcript expression in the silenced samples by qRT-PCR, and compared these levels with samples transfected with scrambled siRNA and untransfected cell lines. We also determined expression levels of *Cyclin D2, Plakoglobin, NKX2.2 *and *PAX6 *by qRT-PCR in silenced, control, and untransfected cell lines. Thereafter, we assessed the expression of these genes in 14 medulloblastoma and astrocytoma cell lines as well as 41 primary tumor samples by qRT-PCR. Transcript expression of every gene was normalized to GAPDH [25]. For qRT-PCR we used iQ™ SYBR^® ^Green Supermix (Bio-Rad, Hercules, CA) as the fluorescence dye to monitor amplification of cDNA. Each sample was run in triplicate and the means and standard deviations were determined. We also compared expression of the candidate genes in our samples with expression in normal human brain tissue. Reaction conditions for qRT-PCR were 95°C for 10 min, followed by 35 cycles of 95°C (denaturation) for 1 min, 57.4°C to 62°C (annealing temperature, depending on the gene amplified) for 1 min, and 72°C (extension) for 50 s. Further, melting curve analysis was carried out at 72°C for 1 min, and 95°C for 10 mins.

### Western blotting

Proteins were extracted from 8 astrocytoma cell lines and 12 primary astrocytic tumor samples (randomly selected) using the RIPA buffer (0.5% sodium deoxycolate, 0.1% SDS, 1% NP40, in 1× PBS supplemented with protease inhibitor cocktail and PMSF). We were unable to extract protein from medulloblastoma samples due to shortage of tumor samples. A total of 20-30 μg of protein per sample was loaded on a gel for western blotting. The resolved proteins were transferred onto a nitrocellulose membrane (Bio-Rad, Hercules, CA, USA). The membrane was blocked with 5% non-fat milk in TBST buffer to prevent nonspecific binding. The membrane was exposed to the primary antibody: anti-GLI1 (goat polyclonal (N-19); Santa Crux, CA, USA) and anti-Cyclin D2 (rabbit polyclonal (H-289) Santa Cruz, CA, USA) antibodies at 1:400 and 1:200 dilution, respectively in 5% skim milk in TBST overnight at 4°C. The membranes were subsequently exposed to HRP-conjugated secondary antibodies (antigoat IgG and anti-rabbit IgG at a dilution of 1:5000 in TBST) and incubated for 1-2 h at room temperature. Positive interaction of the antibodies with the target protein was detected by enhanced chemiluminescence (ECLTM Western Blotting Analysis System, Amersham Biosciences, Piscataway, NJ, USA) and autoradiography.

### Epigenetic studies of *Cyclin D2 *and *PTCH1 *promoters

Treatment of cells with 5-aza-2'-deoxycytidine and TSA were undertaken according to our previous protocol [26]. Bisulfite modification of genomic DNA extracted from the cell lines and tumor samples was performed using the CpGenome DNA modification Kit S7820 (Chemicon International).

### *Cyclin D2 *promoter methylation analysis

We identified three putative CpG-rich promoter regions [27]. The first CpG island ranges from -1550 to -1288 upstream of the start site (262 bp), the second from -1230 to -982 (248 bp), and the third from -296 to -104 (192 bp). These three regions contain consensus binding sequences for several transcription factors including AP2, PUF, STF, PEA3, E2F and PEA3 [27]. The consensus sequence (GCTCTGCTCGCCCACCACCCAATCCTCGCCTC) for binding of *GLI1 *onto the *Cyclin D2 *promoter region is present in the third CpG island.

We analyzed the *Cyclin D2 *promoter by two independent methods. The first was MCA-Meth [24] (a 108 bp product was amplified from the first CpG region: from -1462 to -1354). The second method was MSP-PCR (primer pairs for this method were taken from a previously published article [28]). Unmethylated primers amplified the region from -1616 to -1394 (first CpG -rich region) giving a product of 222 bp; methylated primers amplified the region from -1394 to -1152 (covering the first and second CpG rich regions) producing a product of 276 bp.

### *PTCH1 *promoter methylation analysis

We analyzed the promoter region of *PTCH1 *from -238 to +62 bp [29], which corresponds with exon 1B [30]. To assess methylation of the *PTCH1 *promoter, we followed the MCA-MSP method [24]. This method gives us two melting peaks which are dependent on amplification with unmethylated or methylated primers.

### Statistical analysis

Statistical analyses were performed using Graphpad Prism 4 (Graphpad Software Inc., San Diego, CA). Data graphed with error bars represent mean and SD from experiments performed in triplicate, unless otherwise noted. Fisher's exact test was used to determine the significance of any difference.

## Results

### Expression of *GLI1 *target genes after siRNA-mediated *GLI1 *silencing

**Figure 1:**
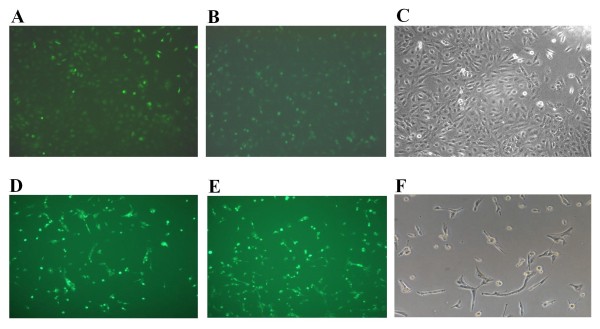
**Transfection of Daoy medulloblastoma cell line and U87MG astrocytoma cell line with *GLI1 *siRNA**. **Figure 1A**: Daoy cell line is transfected with Stealth GLI1 siRNA (Invitrogen) with 100 pmol concentration, using Lipofectamine™ 2000 as a transfecting agent and fluorescein-labeled oligo (BLOCK-iT™ Fluorescent oligo) for identification of trasfection efficency. **Figure 1B**: Daoy cell line transfected with universal negative (U neg) siRNA. **Figure 1C**: Untransfected Daoy cell line. **Figure 1D**: U87MG cell line is transfected with Stealth GLI1 siRNA (Invitogen) with 100 pmol concentration, using Lipofectamine™ 2000 as a transfecting agent and fluorescein-labeled oligo (BLOCK-iT™ Fluorescent oligo) for identification of trasfection efficency. **Figure 1E**: U87MG cell line transfected with universal negative (U neg) siRNA. **Figure 1F**: Untransfected U87MG cell line.

**Figure 2:**
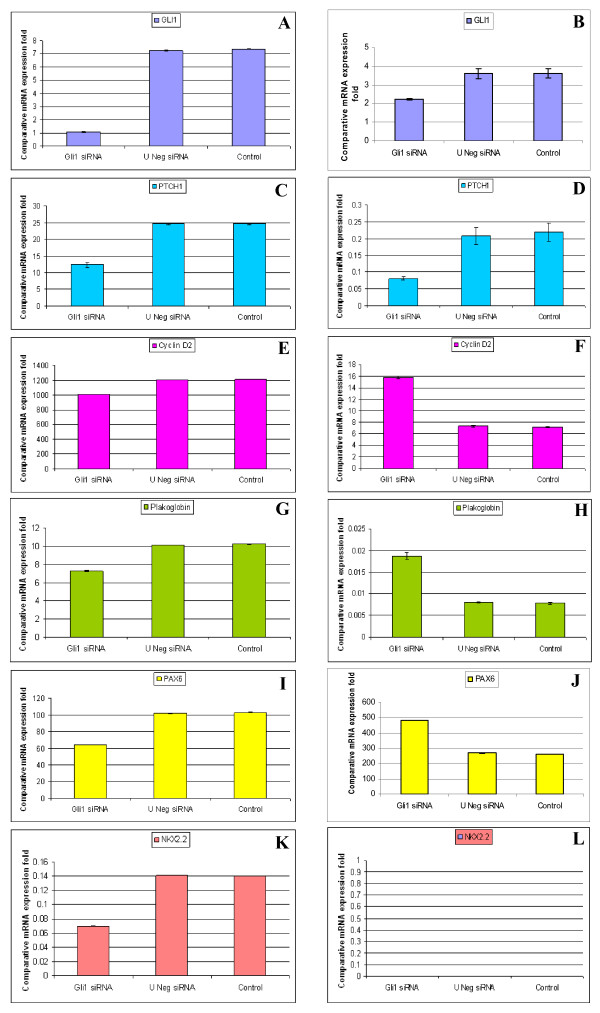
**qRT-PCR comparative expression of *GLI1*, *PTCH1, Cyclin D2, Plakoglobin, PAX6 *and *NKX2.2 *genes in *GLI1 *knock-down, negative and untransfected Daoy medulloblastoma cell line and U87MG astrocytoma cell line**. After 72 h of siRNA-mediated transfection, *GLI1 *showed 83% decrease in expression in Daoy cell line **(Figure 2A) **and 40-45% decrease in expression in U87MG cell line **(Figure 2B) **compared to universal negative siRNA and untransfected cell lines. The siRNA-mediated *GLI1 *knock-down cell lines Daoy and U87MG showed 50% (**Figure 2C**) and 60% (**Figure 2D**) decrease in expression of *PTCH1 *respectively. The *GLI1 *knock-down cell lines (Daoy and U87MG) showed 17% decrease **(Figure 2E) **and 113% increase **(Figure 2F**) in expression of *Cyclin D2 *respectively. The cell lines Daoy and U87MG showed 30% decrease **(Figure 2G) **and 125% increase (**Figure 2H) **in expression of *Plakoglobin *respectively, compared to universal negative siRNA and untransfected cell lines. The knock-down cell lines (Daoy and U87MG) showed 35% decrease **(Figure 2I**) and 100% increase **(Figure 2J) **in expression of *PAX6 *respectively, compared to universal negative and untransfected cell lines. We further checked the expression of *NKX2.2 *in these two *GLI1 *knock-down cell lines (Daoy and U87MG) and found 50% decrease **(Figure 2K) **and not any changes in the expression of *NKX2.2 ***(Figure 2L) **respectively, compared to universal negative and untransfected cell lines.

We transfected Daoy and U87MG cell lines with *GLI1 *siRNA and scrambled siRNA. We monitored the efficiency of transfection using lipofectamine through the delivery of the BLOCK-iT™ Fluorescent Oligo (Figures 1A-F). Images were captured using a fluorescent imaging microscope and similar efficiencies of transfection were attained for both the *GLI1 *siRNA and the scrambled siRNA. To assess efficiency of silencing, expression of *GLI1 *was monitored by RT-PCR after transfection. Approximately 86% and 40-4 5% silencing of *GLI1 *transcript was achieved in Daoy and U87MG cell lines respectively (Figures 2A-B).

Silencing of *GLI1 *resulted in a 50% and 60% decrease in *PTCH1 *expression in Daoy and U87MG cell lines respectively (Figures 2C-D). In contrast, *Cyclin D2 *showed a 17% decrease in Daoy and 113% increase in U87MG cells (Figures 2E-F). *Plakoglobin *expression was decreased by 30% in Daoy cells and increased by 125% in U87MG cells (Figures 2G-H). *PAX6 *expression was decreased by 35% in Daoy cells and increased by 100% in U87MG cells (Figures 2I-J), and *NKX2.2 *was decreased by 50% in Daoy cells and unaltered in U87MG cells (Figures 2K-L). All changes listed were specific to *GLI1 *silenced cells and cells transfected with scrambled siRNA or untransfected cells did not show similar trends.

#### PTCH1

siRNA-mediated silencing of *GLI1 *resulted in an 86% decrease in *GLI1 *transcript and a 50% decrease in *PTCH1 *transcript in the Daoy cell line compared with scrambled siRNA and untransfected Daoy cell lines.

**Figure 3:**
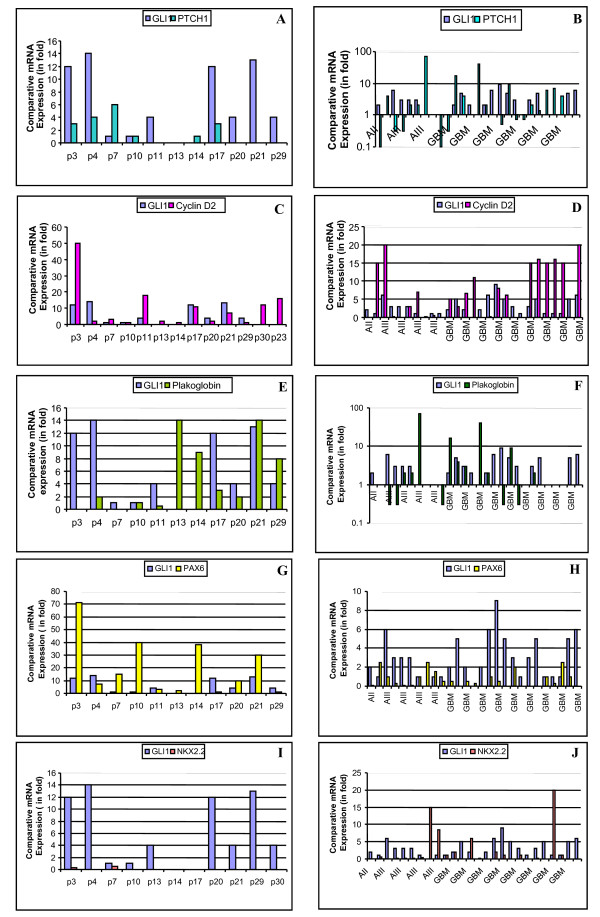
**qRT-PCR comparative expression of *GLI1*, *PTCH1, Cyclin D2, Plakoglobin, PAX6 and NKX2.2 *in medulloblastoma and astrocytic primary tumor samples**. **Figure 3A**: Most of the medulloblastoma primary tumor samples show high fold *GLI1 *transcript expression, but *PTCH1 *expression does not associate. **Figure 3B**: Most of the astrocytic primary tumor samples showed high fold *GLI1 *transcript expression but *PTCH1 *expression is low in most of the primary samples. **Figure 3C**: Most of the medulloblastoma samples show high expression of *GLI1 *and *Cyclin D2. ***Figure 3D**: No distinct pattern of *Cyclin D2 *expression in GBM samples. However, grade III astrocytoma samples show low expression in the presence of high *GLI1 *expression. **Figure 3E**: In case of *Plakoglobin *there is a reverse relationship in expression of *GLI1 *in medulloblastoma except samples 10 and 21. **Figure 3F**: Low-grade astrocytoma samples do not show *Plakoglobin *expression, and high-grade astrocytoma samples show *Plakoglobin *expression in the absence of *GLI1 *expression. **Figure 3G**: Reverse correlation between *GLI1 *and *PAX6 *transcript expression among medulloblastoma samples. **Figure 3H**: In the presence of high *GLI1*, there is low expression of *PAX6 *among astrocytic samples. **Figure 3I**: There is not any expression of *NKX2.2 *among medulloblastoma tumor samples. **Figure 3J**: Most of the astrocytic tumor samples show low/absence of expression of *NKX2.2 *in the presence of *GLI1*.

We subsequently sought to determine the pattern of *PTCH1 *transcript expression in 6 medulloblastoma cell lines and 14 primary medulloblastoma samples and observed that 50% of the cell lines and tumor samples showed high expression of *PTCH1 *[26] (Figure 3A). However, we were unable to find any significant correlation between *GLI1 *and *PTCH1 *expression in the cell lines and tumor samples (p = 0.07).

We also analyzed 27 astrocytoma tumor samples for co-expression of *PTCH1 *and *GLI1. *We observed a mixed expression pattern amongst the low-grade to high-grade samples. Few of the high-grade tumors expressed high levels of *PTCH1 *in the absence of *GLI1 *expression and few of them showed high expression in the presence of *GLI1 *expression. Overall, the pattern of *PTCH1 *expression was low amongst all samples, despite varying levels of *GLI1 *expression (Figure 3B).

#### Cyclin D2

**Figure 4:**
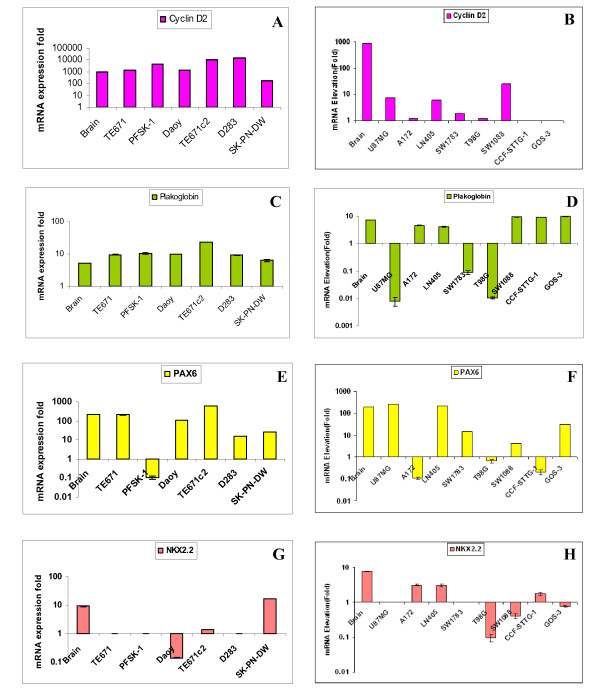
**qRT-PCR comparative expression of *Cyclin D2, Plakoglobin, PAX6 *and *NKX2.2 *in medulloblastoma and astrocytoma cell lines (for GLI1 and PTCH1, please check reference **[26]). **Figure 4A**: All 6 medulloblastoma cell lines show equal expression of *Cyclin D2 *as compared to normal adult brain tissue; interestingly normal brain itself shows high expression of *Cyclin D2*. **Figure 4B**: Four astrocytic cell lines show very low expression and the remaining 4 cell lines show no expression of *Cyclin D2 *transcript as compared to normal adult brain tissue (p < 0.001). **Figure 4C**: All 6 medulloblastoma cell lines show high fold expression of *Plakoglobin *compared to normal adult brain tissue. **Figure 4D**: Five astrocytic cell lines show low expression of *Plakoglobin *transcript compared to normal brain tissue. **Figure 4E**: Only one medulloblastoma cell line, TE671c2, shows high expression fold of *PAX6 *as compared to normal adult brain tissue. **Figure 4F**: Most of the astrocytoma cell lines, except U87MG and LN405, show low expression of *PAX6 *transcript compared to normal brain tissue. **Figure 4G**: Most of the medulloblastoma cell lines show no/low expression of *NKX2.2 *except SK-PN-DW, as compared to normal adult brain tissue. **Figure 4H**: All 8 astrocytic cell lines show no/low expression of *NKX2.2 *transcript compared to normal brain tissue. SYBER Green dye was used for the quantification of cDNA by qRT-PCR. Comparative expression of samples was plotted on Y-axis with Log10.

Silencing *GLI1 *in the Daoy cell line resulted in a 17% decrease in *Cyclin D2 *expression compared with scrambled siRNA and untransfected cell lines (Figure 2E). We evaluated *Cyclin D2 *expression in 6 cell lines and 14 medulloblastoma tumor samples and observed that most of them showed high expression levels of *Cyclin D2*, with the exception of cell line SK-PN-DW (Figure 4A) and one tumor sample (Figure 3C, Table 2). We did not observe any significant correlation between *GLI1 *and *Cyclin D2 *expression.

Six astrocytoma cell lines (U87MG, A172, LN405, SW1783, T98G and SW1783) expressed *Cyclin D2 *at very low levels, and two cell lines (CCF-STTG-1 and GOS-3) did not express *Cyclin D2 *compared to normal adult brain tissue (p = 0.006) (Figure 4B). Fourteen high grade astrocytomas (grades III and IV) were assessed for *Cyclin D2 *and *GLI1 *expression: only 2 glioblastomas (grade IV) co-expressed *Cyclin D2 *and *GLI1 *at high levels (Figure 3D). Low levels of *GLI1 *were found to be associated with high expression levels of *Cyclin D2 *in the glioblastoma samples (p = 0.007).

#### Plakoglobin

We observed a 30% decrease in expression of *Plakoglobin *in upon silencing of *GLI1 *in Daoy transfected cells (Figure 2G). Most of the 6 cell lines and 14 primary tumor samples analyzed showed high expression levels of *Plakoglobin *compared to normal brain tissue (Figure 4C). Additionally, we detected an inverse correlation in levels of expression of *GLI1 *and *Plakoglobin *in primary medulloblastoma samples, with the exception of two tumors (Figure 3E). However, this correlation was not significant.

Among the 8 astrocytic cell lines, 5 (U87MG, A172, LN405, SW1783 and T98G) showed low levels of *Plakoglobin *expression, and the remaining 3 (SW1088, CCF-STTG-1 and GOS3) expressed *Plakoglobin *at levels higher than seen in normal adult brain tissue (p = 0.02) (Figure 4D). A majority of the astrocytic tumor samples (24/27, 89%) expressed *Plakoglobin *at low levels compared to normal brain tissue (Figure 3F). There was a distinct pattern of *Plakoglobin *expression in astrocytic tumor samples: low-grade (AII) samples did not express *Plakoglobin *and few high-grade samples highly expressed *Plakoglobin *in the absence of *GLI1 *mRNA expression. The remaining samples expressed *Plakoglobin *at low levels in the presence of *GLI1*.

#### PAX6

Our results show a 35% reduction in expression of *PAX6 *gene upon *GLI1 *silencing in the Daoy cell line compared with scrambled and untransfected controls (Figure 2I). In our study, we monitored the expression of the *PAX6 *gene in 6 medulloblastoma cell lines and 14 medulloblastoma primary tumor samples: a majority of the cell lines showed moderate expression levels of *PAX6 *with the exception of the PFSK-1 and D283 cell lines (p = 0.015) (Figure 4E). Similarly, most of the primary tumor samples showed high levels of *PAX6 *expression compared to *GLI1 *in the same samples (Figure 3G).

Most of the astrocytic cell lines (A172, SW1783, T98G, SW1088, CCF-STTG-1 and GOS-3) expressed *PAX6 *at low levels, and only 2 (U87MG and LN405) expressed *PAX6 *at high levels compared to normal adult brain tissues (p = 0.006) (Figure 4F). Amongst the primary tumor samples, 20/27 (74%) samples showed low expression of *PAX6 *as compared to normal adult brain tissue (Figure 3H). Interestingly, most of the astrocytic tumor samples showed low expression levels of *PAX6 *even though *GLI1 *was expressed at high levels.

#### NKX2.2

Silencing of *GLI1 *resulted in a decrease in *NKX2.2 *by 50% in the Daoy cell line compared with scrambled siRNA transfected and untransfected cells (Figure 2K). Expression of *NKX2.2 *was low in 2 cell lines (Daoy and TE671c2), high in only one (SK-PN-DW) (p = 0.015) and was not expressed in the remaining 3 (TE671, PFSK-1 and D283) (Figure 4G). Primary medulloblastomas also showed a similar pattern of low expression of *NKX2.2 *and this correlated with high expression levels of *PAX6 *(Figures 3G-I).

Six astrocytic cell lines (A172, LN405, T98G, SW1088, CCF-STTG-1 and GOS-3) showed very low expression of *NKX2.2*, and the remaining 2 cell lines (U87MG and SW178) did not express it at all, compared with expression levels in normal adult brain tissue (p = 0.0001) (Figure 4H). Out of the 27 astrocytic tumor samples, 20 (74%) showed low expression of *NKX2.2 *compared with normal adult brain tissue (p < 0.001) (Figure 3J). Amongst the samples, low-grade tumors did not express *NKX2.2*; however, few of the high-grade samples showed very high expression levels of *NKX2.2 *and low expression of *GLI1*. Overall, most samples expressed *NKX2.2 *at low levels while expressing *GLI1 *to a high degree (Figure 3J).

### No correlation between GLI1 and Cyclin D2 protein expression

**Figure 5:**
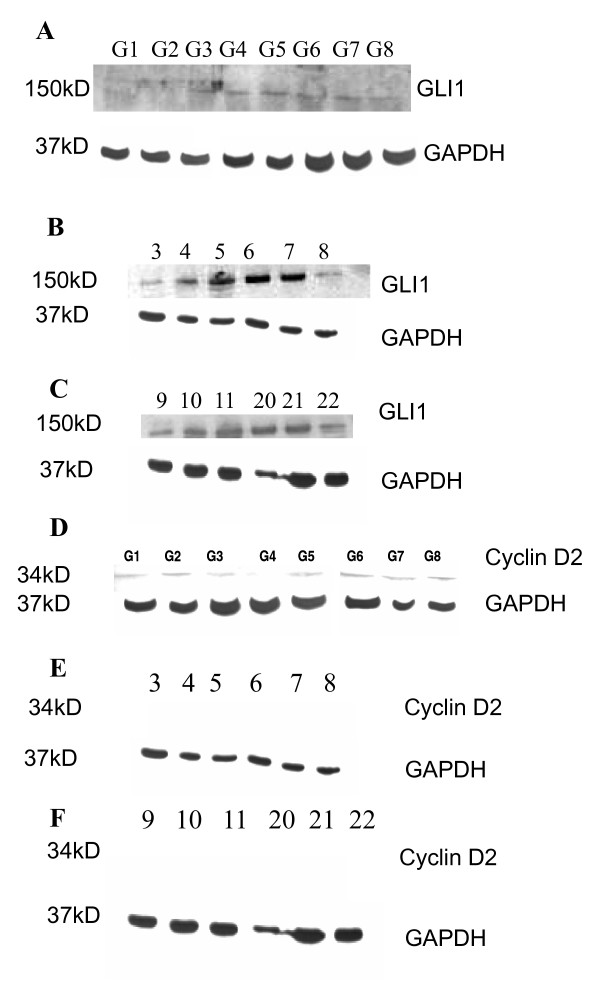
**Protein expression of GLI1 and Cyclin D2 in astrocytic cell lines and tumor samples**. **Figure 5A**: The first two cell lines (G1: U87MG and G2: A172) do not show very distinct bands, but the remaining 6 cell lines (G3: LN405, G4: SW1783, G5: T98G, G6: SW1088, G7: CCF-STTG-1 and G8: GOS-3) show distinct bands of GLI1 protein. **Figures 5B and Figure 5C **show GLI1 protein expression in astrocytic tumor samples 3-8 and 9, 10, 11, 20, 21 and 22. **Figure 5D**: Low protein expression of Cyclin D2 in astrocytic cell lines G1: U87MG, G2: A172, G3: LN405, G5: T98G, G6: SW1088, G7: CCF-STTG-1 and G8: GOS-3. Only one cell line (G4: SW1783) does not express Cyclin D2 protein. **Figures 5E and Figure 5F**: Cyclin D2 protein expression is not shown in astrocytic tumor samples (3-8 and 9, 10, 11, 20, 21 and 22).

GLI1 protein was expressed in most astrocytic cell lines with the exception of two (U87MG and A172) (Figure 5A). However, very low expression of Cyclin D2 protein was observed in the following seven cell lines: U87MG, A172, LN405, SW1088, T98G, CCF-STTG-1 and GOS-3; moreover, there was no expression of Cyclin D2 in one cell line (SW1783) (Figure 5D). Most of the primary tumor samples showed high expression levels of the GLI1 protein (Figures 5B-C) but these samples did not express Cyclin D2 (Figures 5E-F).

### *Cyclin D2 *and *PTCH1 *epigenetics

**Figure 6:**
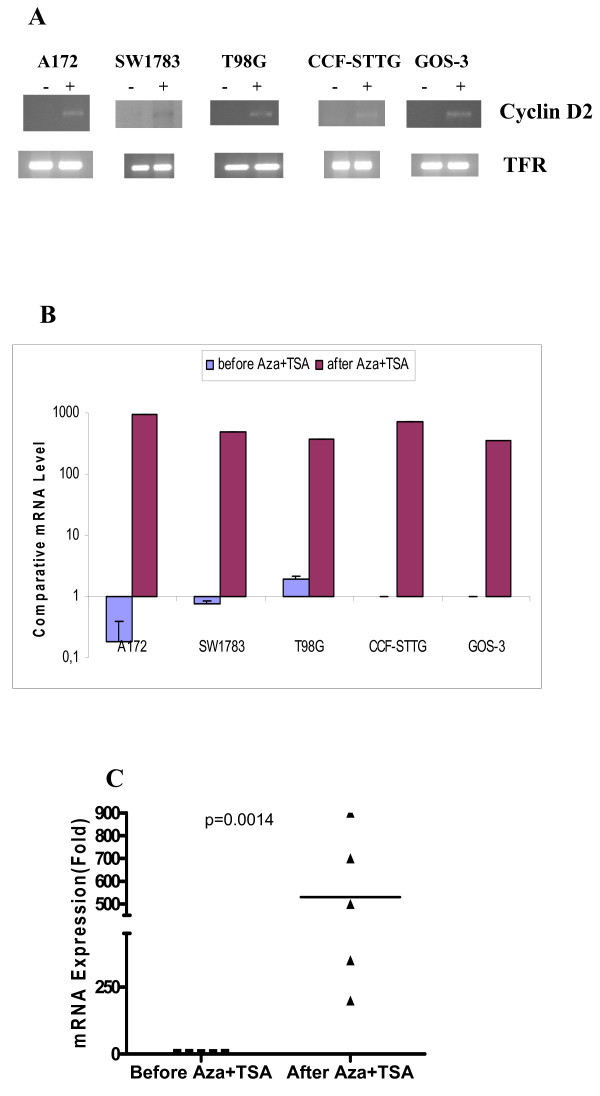
**Reversal of *Cyclin D2 *expression after treatment with demethylating agent 5-Aza-2'-deoxycytidine and TSA**. **Figure 6A**: All 5 cell lines (A172, SW1783, T98G, CCF and GOS-3) show reversal in the expression of *Cyclin D2 *by RT-PCR. -: before treatment; +: after treatment. **Figure 6B**: Reversal of expression of *Cyclin D2 *transcript in the 5 cell lines after the treatment with 5-Aza-2'-dC and TSA, by qRT-PCR. **Figure 6C**: Comparative fold expression of *Cyclin D2 *after the treatment with 5-Aza-2'-dC and TSA. Some astrocytic cell lines even showed 200 fold increase in Cyclin D2 expression (p = 0.0014).

We did not observe any changes in the pattern of *Cyclin D2 *expression upon treatment of the medulloblastoma cell lines with 5-Aza-2'-deoxycytidine and TSA. However, treatment with these compounds resulted in the onset of *Cyclin D2 *expression, assessed by both RT-PCR and qRT-PCR in five astrocytoma cell lines that did not initially express *Cyclin D2 *(A172, SW1783, T98G, CCF-STTG-1 and GOS-3) (Figures 6A-B) (Table 3).

The increase in *Cyclin D2 *mRNA in these cell lines was statistically significant (p = 0.0014) (Figure 6C).

**Figure 7:**
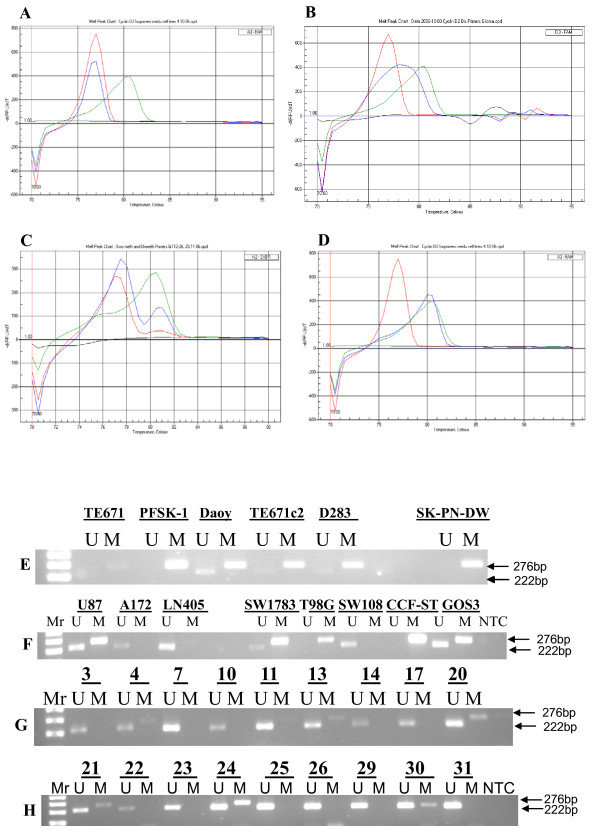
**Promoter methylation analysis of *Cyclin D2***. **7A-D**: Medulloblastoma and astrocytoma cell lines unmethylation melting curve pattern **(Figure 7A); **hemi-methylation curve pattern **(Figure 7B)**; partial methylation curve pattern **(Figure 7C); **and complete methylation curve **(Figure 7D) **of *Cyclin D2 *promoter shown by the MCA-Meth method. **Figure 7E**: *Cyclin D2 *promoter methylation in medulloblastoma cell lines by MSP. Three cell lines (Daoy, TE671c2 and D283) show hemimethylation (U+M) and the remaining three cell lines (TE671, PFSK-1 and SK-PN-DW) show complete methylation (M). Unmethylated and methylated PCR product bands are 222 and 276 bp, respectively. **Figure 7F**: Among astrocytic cell lines three of them (U87MG, SW1783 and GOS-3) show hemi-methylation; 2 show complete methylation (M) (T98G and CCF-STTG-1); and the other 2 (A172 and LN405) show no methylation (U). **Figure 7G**: Promoter methylation analysis of medulloblastoma samples. **Figure 7H**: Promoter methylation analysis of astrocytoma samples.

Amongst the 6 medulloblastoma cell lines assayed using the MCA-Meth method, only PFSK-1 and SK-PN-DW showed a hemi-methylation melting curve (Figure 7B). However, hypermethylation was revealed in all cell lines by MSP (Figure 7E) (Table 1). Methylation associated with low *Cyclin D2 *expression was evident only in the SK-PN-DW cell line (Table 1). In medulloblastoma samples, no methylation was detected by MCA-Meth primers (Figure 7A), even though some tumors expressed *Cyclin D2 *at low levels. However, MSP showed promoter hypermethylation which was associated with the lack of mRNA expression or low expression in tumor samples (Figure 7G) (Table 2).

In astrocytomas, methylation analysis by MCA-Meth and MSP-PCR methods was performed on 8 cell lines. Five of the cell lines did not express *Cyclin D2*, and 3 of these (U87MG, T98G, and GOS-3) showed methylation/partial methylation by MCA-Meth analysis (Figures 7B-C). However, the remaining 2 cell lines did not express *Cyclin D2*, nor did they show any methylation curves by the MCA-Meth method (Figure 7A). However, MSP identified methylation/partial methylation in 6/8 cell lines (Figure 7F). U87MG, which showed partial methylation also expressed *Cyclin D2*, albeit at lower levels compared with normal adult brain tissue (Table 3). We performed the same assays to evaluate *Cyclin D2 *promoter methylation in primary astrocytoma samples and found that the *Cyclin D2 *promoter was methylated/partially methylated in 14/44 (32%) of the tumors (Figures 7B-D) (Table 4). MSP-PCR identified 12/44 (27%) of the samples as methylated at the *Cyclin D2 *promoter (Figure 7H). However, only 4 astrocytic tumor samples (3, 13, 18 and 24) demonstrated *Cyclin D2 *promoter hypermethylation by both methods (Table 4).

**Figure 8:**
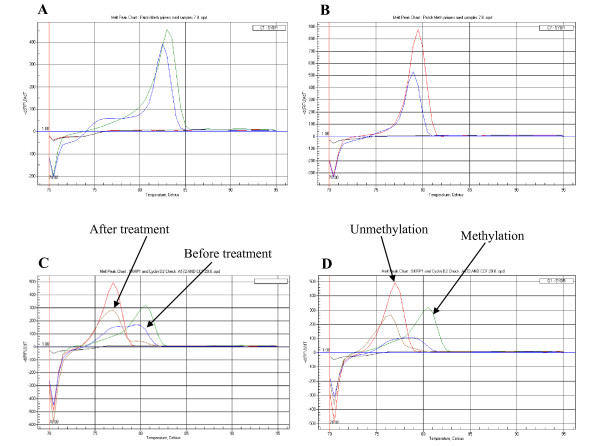
**PTCH1 promoter methylation analysis**. **Figure 8A**: *PTCH1 *promoter methylation curve by MCA-MSP. **Figure 8B**: *PTCH1 *promoter unmethylation curve. Melting curve shown by MCA-Meth. **Figure 8C-D**. Reversal of methylation melting curve after the treatment with 5-Aza-2'-dC and TSA in CCF-STTG-1 **(Figure 8C) **and GOS-3 **(Figure 8D) **astrocytic cell lines.

We also compared the melting curves (using the MCA-Meth method) of the *Cyclin D2 *promoter region before and after treatment with 5-Aza-2'-dexoycytidine and TSA and found a shift in the melting curve from the methylated promoter (81°C) to the unmethylated promoter (78°C) (Figures 8C-D).

Our *PTCH1 *methylation results among the 6 medulloblastoma cell lines identified a methylated peak in only one cell line, D283 (Figure 8A) (Table 1). This was associated with low levels of *PTCH1 *expression. Despite low expression, we were unable to observe methylated/unmethylated peaks in 2 other cell lines (TE671c2 and SK-PN-DW) (Figure 8B). Among medulloblastomas, 2/8 tumor samples showed methylated melting peaks, which were associated with low expression (Table 2). The *PTCH1 *promoter region was methylated as assayed by the MCA-MSP method in only 1/8 astrocytic cell lines (CCF-STTG-1 (Figure 8A) and 3/44 (7%) primary tumor samples. Interestingly, the cell lines and samples showing *PTCH1 *promoter methylation were either glioblastomas (grade IV astrocytoma) or anaplastic astrocytomas (AIII) (Tables 3 and 4).

## Discussion

In the course of our studies, we sought to understand the regulation of certain downstream target genes of the Shh pathway, including *PTCH1, Cyclin D2, Plakoglobin, NKX2.2*, and *PAX6*, in a panel of medulloblastoma and astrocytoma cell lines and tumors. We attempted to explore any putative regulation of these genes by the major transcription factor involved in Shh signaling, *GLI1*, as well as at the epigenetic level.

### PTCH1

After siRNA-mediated *GLI1 *silencing in Daoy and U87MG cell lines, expression of *PTCH1 *decreased, compared with scrambled siRNA-transfected and untransfected cell lines, which may suggest positive regulation of *PTCH1 *by *GLI1 *in medulloblastomas and astrocytomas. We further assessed *PTCH1 *expression in cell lines and samples of both tumor types, and observed that 50% of the samples showed high expression levels of *PTCH1*. This "mixed-pattern" of *PTCH1 *expression amongst samples suggests that there may be *GLI1 *independent regulatory mechanisms, both at the genetic and/or epigenetic level, influencing *PTCH1 *expression. It is important to note that reports have shown *PTCH1 *promoter hypermethylation in several cancers, including medulloblastomas [31,32].

Our previous study demonstrated a correlation between high expression levels of *GLI1 *and *PTCH1 *[26]. However, some of the cell lines and samples expressed low levels of *PTCH1 *in spite of high *GLI1 *levels, which may be suggestive of the fact that those cells are in a different phase of GLI1/PTCH1 interplay. As a putative explanation, high expression of *GLI1 *associated with low expression of *PTCH1 *may indicate a switch-on of Shh signaling (phase 1), trying to exit from a previous resting point -phase 0- (GLI1 low expression/PTCH1 low expression), to advance towards phase 2, a GLI1 high expression/PTCH1 high expression phase characterized by PTCH1 demethylation and expression, to finally reach phase 3 (GLI1 low expression/PTCH1 high expression), equivalent to switching off the Shh signaling process.

For epigenetic studies, we chose the distal region 1C of the *PTCH1 *promoter [29], and found only 1/6 of the medulloblastoma cell lines bearing methylation at the promoter, although two other cell lines also showed low expression levels of *PTCH1*. Among the medulloblastoma samples, 2/8 (25%) showed methylation together with lack of *PTCH1*expression. Several reports have illuminated aspects of *PTCH1 *epigenetic regulation: no methylation has been reported in the proximal promoter region 1B of the *PTCH1 *promoter in primary medulloblastomas, suggesting the possibility of methylation of its distal region, 1C [31]; a knockout mouse tumor model has documented changes in *PTCH1 *expression after treatment with demethylating agents [32]. To follow up on these reports, we decided to further analyze the hypermethylation of *PTCH1 *proximal promoter region, 1B. Our results identified methylation of the promoter region in only 1/8 astrocytoma cell lines and 3/27 (11%) astrocytic tumor samples of high histologic grades.

The *PTCH1 *promoter was hypermethylated in mice tumor models as demonstrated by changes in *PTCH1 *expression after treatment with demethylating agents [32]. However, another study suggests that there is no methylation of the proximal region of the *PTCH1 *promoter, *PTCH1-1B*, although methylation may be concentrated at the distal end of the promoter, or even alternative exon variants, including *PTCH1-1B *and *PTCH1-1C *[29].

### Cyclin D2

To determine whether *GLI1 *regulates *Cyclin D2 *in medulloblastomas, we quantified levels of *Cyclin D2 *transcript upon silencing of *GLI1 *by siRNA in the Daoy medulloblastoma cell line. We observed a decrease in *Cyclin D2 *expression in comparison to controls, which suggests that *GLI1 *may up-regulate *Cyclin D2*, concurring with previous reports showing similar results in *GLI1 *transformed epithelial cells [13]. This evidence is strengthened by the presence of the *GLI1 *consensus binding sequence on the *Cyclin D2 *promoter [13]. To complete this study, we determined the expression of *Cyclin D2 *in 6 medulloblastoma cell lines and 14 tumor samples, and overall, we observed high expression levels of *Cyclin D2 *that correlated with high levels of *GLI1 *expression. These results indicate that *Cyclin D2 *may be positively regulated by *GLI1 *in medulloblastomas.

*GLI1 *silencing in the astrocytic cell line U87MG led to increased *Cyclin D2 *expression in comparison with controls transfected with scrambled siRNA and untransfected cells. Our results suggest that *GLI1 *does not up-regulate *Cyclin D2*. These results do not concur with a previous report showing up-regulation of *Cyclin D2 *in a *GLI1 *transformed epithelial cell line [13]. This discrepancy suggests there may be two regulatory pathways: first, the differential regulatory action of the Shh signal (it may vary in different tissue types); and second, the dual nature of *Cyclin D2*, behaving at times as an oncogene and other times as a tumor suppressor gene [33].

*Cyclin D2 *expression was either low or absent in 8 astrocytic cell lines in comparison with normal brain tissue, although we detected expression of *Cyclin D2 *protein in all these cell lines, with the exception of SW1044. However, even protein expression was very low in comparison with the housekeeping gene *GAPDH*, used as a positive internal control. We detected low expression levels of *Cyclin D2 *transcript in U87MG, A172, LN405, T98G and SW1088 cell lines, which may correlate with low protein expression. Paradoxically, two astrocytic cell lines (CCF-STTG-1 and GOS-3) did not appear to express *Cyclin D2 *transcript, however, low levels of protein expression was detected. This suggests two possibilities: First, early degradation of *Cyclin D2 *mRNA due to a short half-life, and second, the possibility of differential splicing. We failed to detect expression of Cyclin D2 protein in any of the tumor samples.

Exploration of epigenetic regulation of *Cyclin D2 *in medulloblastomas and astrocytomas was motivated by previous studies which had revealed *Cyclin D2 *silencing in cancers such as breast [34], lung [35], and prostate [36], due to promoter hypermethylation.

Our study revealed, to a certain extent, hypermethylation of the *Cyclin D2 *promoter, although methylation did not fully correlate with silencing of expression in medulloblastoma cell lines. Interestingly, the methylation of the promoter in primary tumor samples was associated with low or no expression of *Cyclin D2*.

We treated astrocytic cell lines that did not express *Cyclin D2 *with the demethylating drug 5-Aza-2'-deoxycytidine and the HDAC inhibitor TSA. The combination of these two drugs improves epigenetic modulation [37]. After 72 h of treatment, we were able to observe *Cyclin D2 *expression in these cell lines (p = 0.0014). Our results are contrary to another study [38] that showed high levels of *Cyclin D2 *expression in the astrocytic cell lines, U87MG and T98G, and a decrease in *Cyclin D2 *expression after treatment with the HDAC inhibitor SAHA. Our results suggest that the demethylating agent rather than TSA, is responsible for *Cyclin D2 *re-expression. However, when we treated these two cell lines with only 5-Aza-2'-deoxycytidine, we observed little to no expression of *Cyclin D2*. However, after 5-Aza-2'-deoxycytidine and TSA treatment, there was an increase in expression of *Cyclin D2 *in T98G, but not in U87MG cell line (accompanied by low expression levels of the Cyclin D2 protein).

Despite hemi-methylation of the *Cyclin D2 *promoter in U87MG cells, there was no change in *Cyclin D2 *expression after treatment. We assessed the hypermethylation status of the *Cyclin D2 *promoter in 8 cell lines and 44 astrocytic tumors by studying two putative CpG islands. SW1783 and CCF-STTG-1 cells appeared to be methylated by MSP but not by MCA-Meth. Neither MCA-Meth nor MSP detected methylation in two other astrocytic cell lines (LN405 and SW1088). Moreover, the A172 cell line did not show methylation or even partial methylation at the two CpG sites in spite of no *Cyclin D2 *expression in this cell line. Treatment with 5-Aza-2'-deoxycytidine and TSA induced expression of *Cyclin D2*, indicating that a third CpG island that we did not analyze may play an important role in regulating *Cyclin D2 *expression in this cell line. Unfortunately, we were unable to obtain any primers to assess hypermethylation at this CpG region. Interestingly, a *GLI1 *binding consensus sequence is also located at the third CpG-rich region of *Cyclin D2 *promoter, indicating the possibility of "patches hypermethylation" at this promoter [39] (differential methylation pattern in a promoter region which is supposed to be distributed at CpG rich regions).

### Plakloglobin

We attempted to determine *Plakoglobin *expression in the Daoy medulloblastoma cell line upon *GLI1 *silencing. *Plakoglobin *expression was decreased when compared with control-transfected and untransfected cell lines. This indicates that *GLI1 *may positively regulate *Plakoglobin *expression. However, our results do not concur with a previous report which suggests that *GLI1 *down-regulates *Plakoglobin *in *GLI1 *transformed epithelial cells [13]. Additionally, we assessed *Plakoglobin *expression in 6 medulloblastoma cell lines and 14 tumor samples. A majority of the cell lines and tumor samples displayed high expression levels of *Plakoglobin*, while only a few of the tumor samples showed little or no expression of *Plakoglobin*. Notably, one report suggests that high expression of *Plakoglobin *in medulloblastoma samples is considered to be of high prognostic value [40]. Therefore, our results may support high expression and up-regulation of *Plakoglobin *by *GLI1 *in medulloblastomas.

High levels of *Plakoglobin *expression after *GLI1 *silencing in the U87MG astrocytoma cell line is not indicative of positive regulation of this gene by *GLI1*. This result concurs with a previous study on *GLI1*-transformed epithelial cells [13]. We then sought to determine *Plakoglobin *expression in 8 astrocytic cell lines and 23 primary astrocytic tumor samples. More than 60% (5/8) of the cell lines and 89% (24/27) of the tumor samples expressed *Plakoglobin *at lower levels than normal adult brain tissue. Interestingly, there was a distinct pattern of *Plakoglobin *expression amongst astrocytic tumor samples: low-grade samples expressed *Plakoglobin*, while a few high-grade samples also showed high expression levels of *Plakoglobin *in absence of *GLI1 *transcript. However, the remaining samples all showed low levels of *Plakoglobin *expression in presence of *GLI1 *transcript. These results support that *GLI1 *does not appear to up-regulate *Plakoglobin *in astrocytomas.

### PAX6

Silencing of *GLI1 *in Daoy cells indicated that *GLI1 *may up-regulate the expression of the homeodomain transcription factor I *PAX6 *in medulloblastomas. We also determined the expression of *PAX6 *in 6 medulloblastoma cell lines and 14 primary tumor samples and observed all cell lines with the exception of two expressed high levels of *PAX6*. Similarly, a majority of the primary tumor samples expressed high levels of *PAX6 *transcript compared to normal brain tissue. Previous studies have shown that *GLI1 *down-regulates *PAX6 *gene expression during normal neuronal development [14,41,42]. *PAX6 *is a transcription factor which regulates several genes involved in cell fate, proliferation, as well as migration of neuroectodermal precursor cells during development [43,44]. Interestingly, this suggests different mechanisms of Shh regulation during normal and malignant tissue development. A few studies report high expression levels of *PAX6 *in medulloblastoma samples [45]. Due to the multifunctional roles of this group of genes, it is entirely possible that other mechanisms regulating *PAX6 *in medulloblastomas exist, which further need to be explored.

Subsequent to *GLI1 *silencing, we observed an increase in *PAX6 *expression in the transfected astrocytoma cell line U87MG. A majority of the cell lines displayed low levels of *PAX6 *despite high *GLI1 *expression, as was similarly seen in primary astrocytic tumor samples. Thus, *GLI1 *does not appear to up-regulate *PAX6 *expression in astrocytic tumors.

### NKX2.2

*GLI1 *silencing suggests that *GLI1 *may up-regulate the homeodomain transcription factor II *NKX2.2*. in medulloblastomas. We failed to detect expression of *NKX2.2 *transcript in 3 cell lines, observed low expression in 2, and high expression in only one cell line. This pattern of *NKX2.2 *transcript expression was recapitulated in tumor samples, and was associated with high levels of *PAX6 *transcript in cell lines and tumors.

*GLI1 *silencing did not perturb *NKX2.2 *expression in the astrocytic cell line U87MG. A majority of astrocytic cell lines (75%) and astrocytoma samples (70%) showed either low or no expression of *NKX2.2 *compared to normal adult brain tissue. Nevertheless, a few high-grade samples expressed *NKX2.2 *at very high levels when *GLI1 *was expressed at low levels. Overall, a majority of the samples displayed low levels of *NKX2.2 *expression in the presence of high *GLI1 *expression. Interestingly, however, reports suggest that Shh signaling up-regulates *NKX2.2 *expression during normal neuronal development [42,46]. Low expression levels of *NKX2.2 *seen in our study, despite active Shh signaling, is suggestive of differential Shh signaling during normal development and in astrocytomas. Our study further supports a previous report [47] which shows that *NKX2.2 *is a direct target gene of Shh signaling, and is up-regulated during normal development.

### Statistical analysis

Statistical analysis by the Fisher's test revealed significant correlations of *GLI1 *expression with PAX6 (p = 0.015) and NKX2.2 (p = 0.015) expression in medulloblastoma cell lines. However, we did not find significant correlations in the expression of *GLI1 *with *PTCH1*, *Cyclin D2 *or *Plakoglobin *in the medulloblastoma cell lines. Similarly, in medulloblastoma primary tumor samples, only expression of *NKX2.2 *showed significant correlation with *GLI1 *expression (p = 0.004).

On the contrary, we observed significant correlation of *GLI1 *expression with downstream target genes *PTCH1 *(p = 0.07), *Cyclin D2 *(p = 0.006), *Plakoglobin *(p = 0.02), *PAX6 *(p = 0.006) and *NKX.2.2 *(p = 0.0001) in astrocytoma cell lines. Finally, *GLI1 *expression correlated significantly with downstream target genes *PTCH1 *(p = 0.005), *Cyclin D2 *(p = 0.04), *Plakoglobin *(p = 0.006), *PAX6 *(p = 0.002) and *NKX2.2 *(p = 0.008) in astrocytoma primary tumor samples.

## Conclusions

We report that *Cyclin D2 *and *PTCH1 *are regulated by two mechanisms: at the transcriptional level and at the epigenetic level. *GLI1 *appears to up-regulate *PTCH1 *expression in both medulloblastomas and astrocytomas, and remaining genes tested, namely, *Cyclin D2, Plakoglobin, PAX6*, and *NKX2.2*, only in medulloblastomas. Analysis of promoter methylation suggests that epigenetic regulation of *Cyclin D2 *is stronger in astrocytomas than in medulloblastomas, while epigenetic regulation of *PTCH1 *is weak in both tumors. Based on our results, we advocate that molecules that inhibit Shh activation as well as epigenetic modulator drugs may be effectively used for the treatment of astrocytoma tumors.

## Abbreviations

MSP: Methylation specific PCR; PCR: Polymerase Chain Reaction; MCA-Meth: Melting Curve Analysis-based methylation assay; MCA-MSP: Melting Curve Analysis-based Methylation Specific PCR; qMSP: Quantitative Methylation Specific PCR; qRT-PCR: Quantitative (real time) Reverse Transcribed-PCR; Shh: Sonic hedgehog; 5-Aza-2'-dC: 5'-Aza-2'-deoxycytidine; HDAC: Histone Deacetylase Inhibitor; TSA: Trichostatin A; C_T_: threshold cycle; RPMI Medium: Roswell Park Memorial Institute Medium; IMD: in vitro methylated DNA

## Competing interests

The authors declare that they have no competing interests.

## Authors' contributions

MHS carried out siRNA transfection, PCR standardization, samples processing, western blot, and Promoter Methylation studies. JSC, XF, JAR, CGE, MA and SS conceived the study, assisted in study design, statistical analysis, coordinated and helped in the draft of the manuscript. All the authors have read and approved the final version of the manuscript.

## Pre-publication history

The pre-publication history for this paper can be accessed here:

http://www.biomedcentral.com/1471-2407/10/614/prepub
